# DNA Barcoding for Community Ecology - How to Tackle a Hyperdiverse, Mostly Undescribed Melanesian Fauna

**DOI:** 10.1371/journal.pone.0028832

**Published:** 2012-01-13

**Authors:** Rene Tänzler, Katayo Sagata, Suriani Surbakti, Michael Balke, Alexander Riedel

**Affiliations:** 1 Department of Entomology, Zoologische Staatssammlung, Munich, Germany; 2 Papua New Guinea Institute for Biological Research, Goroka, Papua New Guinea; 3 Department of Biology, Universitas Cendrawasih, Jayapura, Indonesia; 4 GeoBioCenter, Ludwig-Maximilians-University, Munich, Germany; 5 Department of Entomology, Staatliches Museum für Naturkunde, Karlsruhe, Germany; Biodiversity Insitute of Ontario - University of Guelph, Canada

## Abstract

**Background:**

*Trigonopterus* weevils are widely distributed throughout Melanesia and hyperdiverse in New Guinea. They are a dominant feature in natural forests, with narrow altitudinal zonation. Their use in community ecology has been precluded by the “taxonomic impediment”.

**Methodology/Principal Findings:**

We sampled >6,500 specimens from seven areas across New Guinea; 1,002 specimens assigned to 270 morphospecies were DNA sequenced. Objective clustering of a refined dataset (excluding nine cryptic species) at 3% threshold revealed 324 genetic clusters (DNA group count relative to number of morphospecies = 20.0% overestimation of species diversity, or 120.0% agreement) and 85.6% taxonomic accuracy (the proportion of DNA groups that “perfectly” agree with morphology-based species hypotheses). Agreement and accuracy were best at an 8% threshold. GMYC analysis revealed 328 entities (21.5% overestimation) with 227 perfect GMYC entities (84.1% taxonomic accuracy). Both methods outperform the parataxonomist (19% underestimation; 31.6% taxonomic accuracy). The number of species found in more than one sampling area was highest in the Eastern Highlands and Huon (Sørensen similarity index 0.07, 4 shared species); ⅓ of all areas had no species overlap. Success rates of DNA barcoding methods were lowest when species showed a pronounced geographical structure. In general, *Trigonopterus* show high α and β-diversity across New Guinea.

**Conclusions/Significance:**

DNA barcoding is an excellent tool for biodiversity surveys but success rates might drop when closer localities are included. Hyperdiverse *Trigonopterus* are a useful taxon for evaluating forest remnants in Melanesia, allowing finer-grained analyses than would be possible with vertebrate taxa commonly used to date. Our protocol should help establish other groups of hyperdiverse fauna as target taxa for community ecology. Sequencing delivers objective data on taxa of incredible diversity but mostly without a solid taxonomic foundation and should help pave the road for the eventual formal naming of new species.

## Introduction

Community ecology and conservation biology are highly relevant in these times of global climate change and biodiversity crisis. They rely heavily on species-based hypotheses as their basic currency. Ecologists are increasingly aware of the great importance and added value of “good” taxonomy, e.g., in studies of local species richness, large-scale patterns of biodiversity and regional / global species estimation (α-, β- and γ-diversity) [1]. In particular, comparative studies with large numbers of species would benefit from deeper involvement of taxonomists [2] or from the application of sound taxonomic resources [1]; however, the lack of the latter and the steady decrease of the former hamper species-based research, especially in studies of hyperdiverse arthropods in tropical ecosystems [3]. These highly diverse groups are thus neglected by those setting conservation priorities [4].

Here, we provide a working example using hyperdiverse Melanesian fauna, with a focus on the island of New Guinea. Many great naturalists have studied Melanesian fauna and have sought to explain its extraordinary diversity (e.g., [5], [6], [7]). Recently, community ecologists have established one of the most extensive research programs on large-scale patterns of arthropod diversity, with extensive involvement of taxonomists and parataxonomists and university training of in-country partners [8]. To uncover general patterns in such studies, as many taxonomic groups as possible should be considered. Even when relying on taxonomic expertise, the potentially large number of species poses the question of how to substantiate species-level data. Here, we summarise the first steps necessary for establishing a new taxon in a community ecology research program, building on both DNA sequencing and taxonomic expertise.

We have previously shown that the New Guinea weevils in the genus *Trigonopterus* are locally very diverse, with more than 50 species in a single small mountain range. These species exhibit strong altitudinal zonation and are well characterised by deep divergences in the DNA barcoding fragment of the *cox1* gene [9]. Literature data indicate a wider geographic distribution of *Trigonopterus* in the region; the group can thus potentially serve as an indicator in community ecology research, particularly if (1) species diversity is high across a wider area, (2) there are good morphological characters that can be used to identify specimens by genus and species for future taxonomic work, and (3) the species have generally clear genetic signatures, even when sampling density is very high. One criticism of DNA sequence-based approaches is that genetic signatures might become obscured when sampling is expanded such that it introduces both higher intraspecific variation and sister species, especially recent ones [10], [11]. Most studies on rich tropical fauna were conducted at local scales [9], [12], [13], thus avoiding this problem. Relatively few studies have included the effects of β-diversity and studied a given taxon over a wider geographic range, e.g. [14], [15], [16], [17], [18].

**Figure 1:**
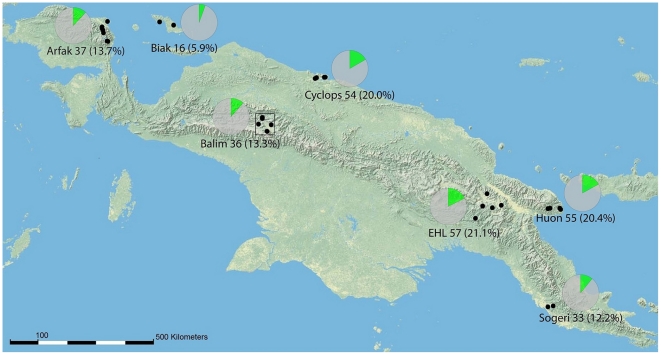
The seven sampling areas across New Guinea. The number of species found at each area is given, with percentage of the total species number in parentheses. Pie charts show the number of species found in the area (green) in relation to New Guinea γ diversity (grey). The results rely on the refined dataset (989 individuals and 270 morphospecies). EHL = Eastern Highlands. The frame in Balim outlines the area shown in the detailed map (see Additional Fig. S1).

We conducted an extensive sampling program at seven sites across the island of New Guinea to investigate the effects of sampling on species diversity patterns. Three different geographical scales were examined: local (0–5 km), mid-range (7–107 km), and distant (160–1700 km, average 850 km) across distinct geological terranes. (Fig. 1). This is also the first study to contrast the error rates of taxonomic sorting, sorting by parataxonomists, and various approaches to DNA-based species delineation. The usefulness of *cox1* sequence data for sustainably enhancing community ecology studies is discussed.

## Materials and Methods

### Diagnostic characters of *Trigonopterus*

For morphological characterisation and the taxonomic background, see http://species-id.net/wiki/Trigonopterus. The apomorphic characters for diagnosis were as follows: 1) complete loss of the metanepisternum, 2) minute tarsal claws, 3) articulation of fourth tarsomere deeply incavated.

### Field methods

The methods for collection and preparation of specimens were previously described by Riedel et al. [9]. The specimens were collected during 2006 and 2010 in seven areas of New Guinea, each of which represents a distinct geological terrane [19] (Fig. 1). As many localities as possible were sampled in each area by collecting along elevational transects and applying different collecting techniques, such as beating (for foliage-frequenting species) and sifting (for edaphic species). The time spent and the number of litter samples taken in each area were recorded (see Table S1). Approximately 6,500 specimens of *Trigonopterus* were collected and screened for this study.

### Selection of specimens and the initial morphospecies hypotheses

**Figure 2:**
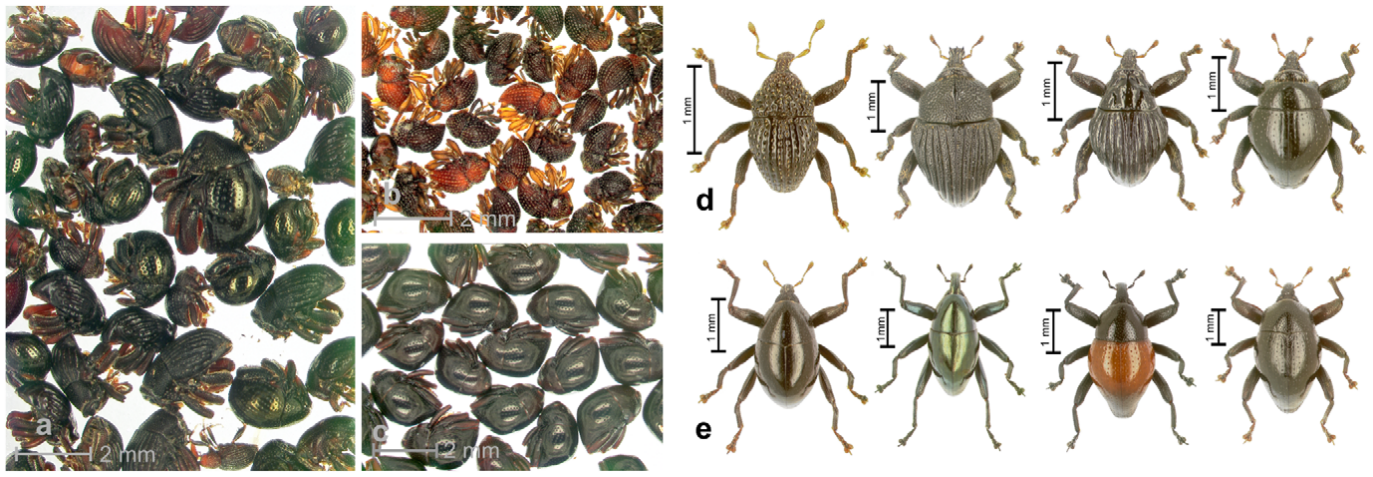
Process of sorting specimens. a) unsorted sample of edaphic weevils including *Trigonopterus* and other genera in ethanol. b–c) sorted samples containing each “initial morphospecies” of *Trigonopterus*. d–e) Dry-mounted specimens of *Trigonopterus* after DNA extraction and the preparation of genitalia; “refined morphospecies”; examples of four characteristic edaphic species (d) and foliage-frequenting species (e).

Specimens from each sampling area were pre-sorted wet in ethanol-filled petri dishes by an expert taxonomist (A. Riedel) to prevent excessive sequencing of the same species. When possible, three to four specimens of each morphospecies were included. External characters such as size, surface sculpture, dorsal outline, and colour were used for the pre-selection (Figs. 2A–C), with the underlying species concept being biological, and a certain degree of morphological difference a (subjective) indicator of potential reproductive isolation. Where possible, specimens were chosen from the most distant localities, e.g., the lowest and highest elevation in the area. Single specimens of uncertain morphospecies assignment were also selected for extraction. No attempt was made to identify identical species across different sampling areas. Male genital characters could not be referred to at this stage. Only unique female specimens that could be identified based on their distinct external morphological characters were included in the final analysis. Altogether, 1,002 *Trigonopterus* were selected for DNA extraction, plus seven outgroup representatives. The initial morphospecies identifications were used in a separate dataset.

### Refined morphospecies hypotheses

After extraction, the preliminary morphospecies hypotheses were revised based on examinations of the male genital characters and dry-mounted specimens (Figs. 2D–E). The judgement of species status was based exclusively on morphological evidence. These refined morphospecies were used in a separate dataset and formed the basis for most subsequent analyses of morphospecies. Our null hypothesis of species was based on morphology.

### Parataxonomist sorting

We made an attempt to contrast the taxonomist's species hypotheses with those of a trained layman, i.e., the parataxonomist. For that purpose, we used our dry-mounted voucher-specimens, genital dissections and attached identification labels hidden. Michael Balke, a water beetle taxonomist who was unfamiliar with weevil classification, was the “highly trained” parataxonomist. Sorting was conducted under highly favourable conditions, as sorting through wet samples in ethanol would be much more difficult; replication of the sorting by other parataxonomists was beyond the scope of this work.

### DNA sequencing

Whole beetles were non-destructively extracted with DNeasy (Qiagen, Hilden, Germany) and NucleoSpin 96 Tissue Kits (Macherey-Nagel, Düren, Germany). We amplified the 5′ end of cytochrome *c* oxidase 1 (*cox1*) for all specimens using primers adjusted for Cryptorhynchinae [20] (see Table S2). For PCR (Mango-Taq, Bioline), we used a two-stage protocol (5 cycles at a 47°C annealing temperature followed by 30 cycles at 52°C [21]. For a few presumably cryptic species and for comparative purposes with few other species, the additional nuclear protein-coding genes arginine kinase [22], histone 4 [23] and elongation factor 1α [24], [25] were amplified and sequenced (see Table S2). The sequences were edited using Sequencher 4.10.1 (GeneCodes Corp., Ann Arbor, MI, USA) and submitted to ENA, European Nucleotide Archive [HE613858–613921; 615156–616164].

### Sequence data analysis, molecular species hypotheses

Sequences were aligned with ClustalW (reference), and maximum likelihood (ML) trees were inferred using raxmlGUI 0.93 [26], [27] with default settings (ML+rapid bootstrap, 200 bootstrap replicates and model GTR+GAMMA). For species delineation, we used two methods: objective clustering [10] and the general mixed Yule coalescent (GMYC) model-based method [28] (see [17]). Objective clustering in SpeciesIdentifier [10] uses uncorrected p-distances to cluster sequences at different thresholds that are preset by the user. SpeciesIdentifier can distinguish which sequences belong to which *a priori*-identified morphospecies, and can provide outputs that allow the calculation of the number of clusters in agreement with existing taxonomy, as well as numbers of lumped and split clusters (see [17]). Neither the use of mean interspecific distances, nor K2P distances are appropriate in barcoding studies [29], [30]. The GMYC [28], [31] approach does not rely on preset thresholds but on information contained in the data itself. GMYC analyses were conducted with “SPLITS” (Species Limits by Threshold Statistics) (http://r-forge.r-project.org/projects/splits) in R Version 2.12.1 [32]. As identical haplotypes included in the dataset are problematic for GMYC, they were removed using Collapse 1.2 [33], resulting in a dataset of 824 658-bp sequences. GMYC requires the calculation of an ultrametric tree, but the tree does not have to be time calibrated. The ultrametric tree was obtained in BEAST version 1.4.7 [34]. The model that best fit the data was GTR+I+G, but as there are concerns relating to the simultaneous use of invariants and gamma distributions in evolutionary models [27], we decided to use the GTR+G model; GRT+G was the second-best model in jModeltest [35]. The Likelihood Ratio Test in DAMBE [36] demonstrated a p<0.001 suggesting the rejection of the null hypotheses (strict clock constraint) and use of the relaxed (uncorrelated log-normal) clock constraint. A coalescent model with a constant population size has been implemented. We obtained 36 M generations by merging nine separate runs and sampling every 1,000th in LogCombiner 1.4.7. After the removal of 1.5 M generations of burnin in each run, the remaining 22,509 trees were analysed with TreeAnnotater version 1.4.7 (http://beast.bio.ed.ac.uk). For the GMYC analysis, we used the single method with standard parameters (interval = c(0,10)) because changing the upper and lower limit of scaling parameters had no noticeable effect on our results (see the SPLITS help manual).

To demonstrate the differences between clustering localities separately and clustering the combined localities of an entire area we used a 95% confidence interval obtained from the seven values for the localities at each clustering threshold to check whether the number of clusters found by SpeciesIdentifier differed significantly from that found for the complete dataset, i.e., whether they fell within the 95% confidence interval. For comparison, we used relative values, calculating percentages out of the absolute number of clusters found for each of the 12 clustering thresholds.

### Final species hypotheses

**Figure 3:**
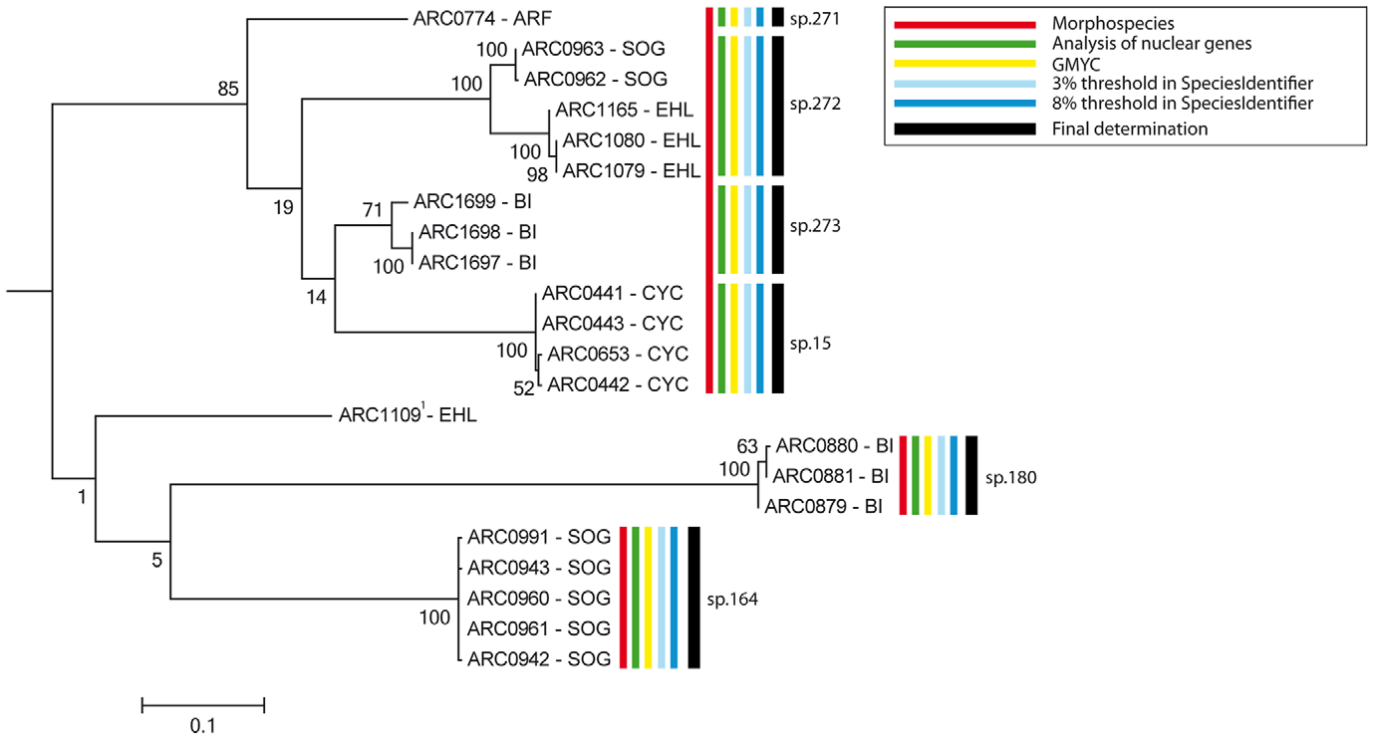
Cox1-based RaxML subtree including three morphospecies for which we sequenced nDNA markers. Red bar = initial morphospecies hypotheses. Additional bars are species delineation based on nDNA markers (green), GMYC analysis (yellow), clustering at different thresholds using SpeciesIdentifier (blue bars) and the final identification based both on morphological and molecular data (black). Bootstrap values at the nodes are based on 200 replicates. In tip labels, areas where specimens were found are indicated: ARF = Arfak, SOG = Sogeri, EHL = Eastern highlands, BI = Biak, CYC = Cyclops. ^1^ ARC1109: single female not included in the analyses.

These were arrived at by studying the distribution of refined morphospecies hypotheses among the molecular entities (Fig. 3; Table 1). Morphospecies with high *cox1* divergence were examined morphologically a second time (see Table S3), and nuclear DNA markers were sequenced to discover potentially diagnostic nDNA characteristics that suggest the existence of “cryptic” species or reveal overlooked species. The final hypotheses incorporate evidence from both morphology and molecules. To avoid circularity, the final species hypotheses were used only for comparison with data derived from the “refined morphospecies”.

**Table 1:** *p*-distances of 40 specimens, partly representing cryptic species.

refined morphospecies hypotheses	% *p*-distance mtDNA	% p-distance nDNA	final morphospecies hypotheses	% *p*-distance mtDNA	% *p*-distance nDNA	Area
sp. 1, *T.* cf. *illex*	20.00 (21.61)	1.17 (9.70)	sp. 1 (N = 5)	0.76 (19.66)	0.07 (8.90)	Cyclops
sp. 1, *T.* cf. *illex*			*sp. 270 (N = 3)	4.86 (21.95)	n/a	Huon
sp. 1, *T.* cf. *illex*			*sp. 275 (N = 1)	n/a (19.49)	n/a (9.04)	EHL
sp. 1, *T.* cf. *illex*			*sp. 276 (N = 3)	0.99 (21.29)	0.73 (4.35)	EHL
sp. 15	8.80 (18.07)	1.80 (10.69)	sp. 15 (N = 4)	0.30 (16.60)	0.62 (7.98)	Cyclops
sp. 15			*sp. 271 (N = 1)	n/a (16.81)	n/a (10.95)	Arfak
sp. 15			*sp. 272 (N = 4)	2.47 (18.69)	0.20 (6.35)	Sogeri & EHL
sp. 15			*sp. 273 (N = 3)	1.98 (16.43)	0.05 (7.08)	Biak
sp. 86	14.21( 18.69)	n/a	sp. 86 (N = 3)	0.15 (19.84)	n/a	Balim
sp. 86			*sp. 274 (N = 1)	n/a (18.46)	n/a	Balim
sp. 150	11.96 (19.68)	n/a	sp. 150 (N = 7)	4.21 (18.27)	n/a	Sogeri & Huon
sp. 150			*sp. 269 (N = 1)	n/a (18.29)	n/a	Huon
sp. 247	7.09 (20.42)	n/a	sp.247 (N = 3)	0.30 (21.15)	n/a	Arfak
sp. 247			*sp.279 (N = 1)	n/a (19.40)	n/a	Arfak

Average intraspecific and interspecific (in parentheses) p-distances for mitochondrial and nuclear DNA. All codon positions are included. All positions containing missing data were eliminated for pairwise sequence comparisons (pairwise deletion option). There were a total of 1275 positions (AK, EF1a and H4) in the nuclear dataset and 658 positions in the mitochondrial dataset. The presence of n/a in the results denotes cases in which it was not possible to estimate evolutionary distances.

*Finally accepted cryptic species. EHL = Eastern Highlands.

### β-diversity

To quantify between-site (β) diversity, the Sørensen similarity index [37] was calculated for each pair among the seven locations as QS = 2 C/A+B, where A and B were the number of species in the two compared localities and C was the number of species shared by the two compared locations.

### Objective Clustering

As performance metrics, we calculated the “number of clusters found relative to the number of morphospecies in the dataset (agreement)” and “the number of perfect clusters relative to the number of morphospecies in the dataset (taxonomic accuracy)” [17]. “Agreement” thus compares *numbers* of molecular and traditional taxonomic units, but not their *contents*. That means that in an agreement of 100%, the number of split morphospecies may be balanced by an equivalent number of lumped morphospecies. However, a taxonomic accuracy of 100% is achieved only when all morphospecies are fully congruent with the molecular groups and vice versa. In practice, high agreement means that the number of cox1 clusters or entities reflects species diversity as defined by an expert taxonomist well, and cox1 sequencing delivers a reasonable proxy for a fast, initial measure of diversity in a sample. High agreement does not neccesarily mean that most or all molecular units directly translate into species. Agreement-values exceeding 100% signal overestimation of species numbers, e.g. 120% agreement equals 20% overestimation, whereas 80% agreement equals 20% underestimation.

## Results

The *cox1* (658 bp), AK (662 bp), EF1α (404 bp) and H4 (209 bp) sequences had no indels after alignment. Amino acid translation detected no stop codons or suspiciously common non-synonymous substitutions that could suggest the presence of pseudogenes. EF1α contained one intron, which was removed prior to further analysis.

Maximum likelihood analyses of *cox1* data included 1,002 *Trigonopterus cox1* sequences and seven outgroups. The resulting *cox1* tree (see Fig. S1) revealed 13 singleton clades that contained females without diagnostic morphological characteristics. These females were excluded from the downstream cluster and GMYC analyses as they cannot reliably be diagnosed morphologically. The resulting alignment of 989 *Trigonopterus* sequences contained 274 morphospecies according to the initial morphospecies hypothesis (without male genitalia data), 270 morphospecies according to the refined morphospecies hypothesis (based on dry-mounted specimens and genital characters, but without input from molecular data) and 279 species according to the final hypotheses (including cryptic species). Along with the full datasets (γ-diversity), we also used the refined hypotheses to count species in each of the seven localities (α-diversity).

Although interspecific divergences in the genus (average 19.80%, between 12.06–23.86%) are much deeper than the intraspecific divergences (average 1.28%, between 0.00–19.49%), some species showed conspicuously high intraspecific divergence (see Table S3). Twenty-five of the refined morphospecies showed a divergence above 3%; these species were subjected to additional scrutiny (see [17]). All of these 25 species were re-investigated morphologically, and for some, we aimed to add nuclear markers.

Five of the above species combined high divergence with partial sympatry or, at least, patterns in which genetic distance was negatively correlated with geographic distance (as in sp. 015, see below). One species was polyphyletic (sp. 001 - *T.* cf. *illex*), i.e., split into two clades that were placed at distant positions on the tree and consisted of four divergent clusters (spp. 001, 275, 276, and sp. 270; the latter associated with sp. 263 - *T.* cf. *densatus*). Two others were paraphyletic; sp. 150 - *T.* cf. *vanus* turned out to be the sister of a clade comprising sp. 014 and sp. 269 - *T.* cf. *vanus*, and sp. 002, sp. 086, and sp. 274 formed a triad. One species was monophyletic but was divided into four highly divergent clusters (spp. 015, 271, 271, 273) that showed an unexpected geographic pattern: sp. 015 from Cyclops Mountains and sp. 273 from Biak Island were closely related to sp. 272 from Sogeri and EHL but not to sp. 271 from the Arfak mountains, which are geographically closer to Biak. Finally, specimen ARC0854 showed a divergence of 7.09% from the sympatric specimens of species 247.

For two of the questionable refined morphospecies (spp. 001, 015), the nuclear markers AK, EF1α and H4 were sequenced and compared with the mtDNA clusters (Fig. 3). In all cases, the mtDNA and nDNA clusters were fully congruent. Sequence divergence and/or morphological differences found by re-examination of the specimens suggested the presence of six cryptic species within these two morphospecies (see Table S3), with a mean interspecific nDNA *p*-distance of 7.31% (smallest: 4.35%). For the remaining three questionable species (spp. 086, 150, 247), with distances of 14.21%, 5.41% and 7.09%, respectively, we conducted only morphological re-examination of the specimens, which confirmed three additional cryptic species (*spp. 269, 274, 279) based on inconspicuous differences in the male genitalia. Some of the suggested cryptic species are allopatric, but *sp. 275 and *sp. 276 occur sympatrically in Haia in the EHL area, as do sp. 086 and *sp. 274 in Bokondini in the Balim area, sp. 247 and *sp. 279 in Mokwam in the Arfak area, and sp. 150 and *sp. 269 in Sattelberg and Pindiu, two localities in the Huon area that are separated by less than 30 km. As sp. 150 has a relatively wide distribution, occurring both in the Huon area and in Sogeri, the two species must be considered sympatric in the Huon area.

*Trigonopterus nasutus* (Cyclops Mts.) and *T.* sp. 018 (Arfak Mts.) were initially classified as two species from two geographical areas. *Cox1* divergence is high, averaging 7.46% between our single *T. nasutus* and the specimens of *T.* sp. 018. Nuclear markers were less divergent (average *p*-distance: 0.36%), and in the absence of marked morphological differences, we opted to consider all specimens as representing one species, *Trigonopterus nasutus*, with a range over two geographical terranes and with pronounced geographical haplotype structure.

After recognition of the cryptic species, the final dataset, with 279 species, showed an average intraspecific divergence of 1.04% (refined dataset before 1.28%) ranging from 0.00–11.40%. Interspecific divergence was unchanged.

### Parataxonomist sorting

Lumping species resulted in 27% error between parataxonomist sorting and our final species hypothesis.

### Objective Clustering

First, we clustered the initial dataset with 274 morphospecies at a 3% cutoff. We found 324 clusters (120% agreement, with a 20% overestimation relative to the 274 morphospecies of the dataset) and 145 perfect clusters (53.7% taxonomic accuracy). The best taxonomic accuracy was found at a 4% threshold, with 146 perfect clusters (54.1%), and the best agreement was found at a 9% threshold, with 269 clusters (99.6%).

Second, we clustered the refined dataset (270 morphospecies) at a 3% cutoff. A total of 947 (95.75%) sequences had at least one conspecific sequence, which equates to 42 singletons; 228 (84.44%) morphospecies had valid conspecifics. We found 324 clusters (120% agreement, or 20% overestimation); of these clusters, 231 were perfect (85.56% taxonomic accuracy) (Table 2). The best taxonomic accuracy was found at an 8% threshold, with 247 perfect clusters (91.48%), and the best agreement at a 9% threshold, with 269 clusters (99.63%). In addition, each locality was clustered separately, resulting in 80.6–100% taxonomic accuracy and 100–125.0% agreement at a 3% threshold (Table 2). The overall success rate was lowest within the Balim area (80.6% taxonomic accuracy; 125% agreement). This finding was likely caused by the strongly structured landscape of the sampling area and a critical 7–52 km distance between the localities, which supposedly allows both high species overlap and marked local differentiation. To test this hypothesis, the four main localities sampled in the Balim area were examined individually, resulting in local success rates of 90.9–100% at a 3% threshold; these results corresponded with the values obtained for the other areas (see Table S4).

**Table 2:** Regional and local clustering at a 3% threshold and GMYC of the final dataset.

analyzed dataset	number morpho-species	number cluster/entities	agreement [%]	number perfect fit	taxonomic accuracy [%]	lumped cluster	split cluster
PT sorting	270	226	81.0	88	31.6	79	59
initial	274	324	120	145	53.7	49	130
refined	270	324	120.0	231	85.6	0	93
refined 8%	270	278	103.0	247	91.5	4	27
refined GMYC	270	328	121.5	227	84.1	0	101
Arfak (ref.)	37	40	108.1	34	91.9	0	6
Biak (ref.)	16	16	100.0	16	100.0	0	0
Balim (ref.)	36	45	125.0	29	80.6	0	16
Cyclops (ref.)	54	61	113.0	49	90.7	0	12
Sogeri (ref.)	33	40	121.2	27	81.8	0	13
Huon (ref.)	55	59	107.3	51	92.7	0	8
EHL (ref.)	57	63	110.5	51	89.5	0	12
final 3%	279	324	116.1	242	86.7	0	82
final 8%	279	278	99.6	258	93.1	5	15
final GMYC	279	328	117.6	239	86.4	0	89

Columns from left to right: (1) name of the dataset used; (2) number of morphospecies that each dataset includes; (3) number of clusters / entities found for each dataset; (4) number of clusters found relative to the number of morphospecies (agreement); (5) number of clusters containing all individuals of one species and none of other species; (6) percentage of perfect clusters relative to morphospecies (taxonomic accuracy); (7) number of clusters containing more than one species; (8) number of clusters containing not all individuals of a species. ref. = refined dataset; PT sorting = parataxonomist sorting.

Finally, we clustered the final dataset with 279 species at a 3% threshold. As the sequences of the final dataset are identical to the initial and refined datasets, the total number of clusters was the same. We found 242 perfect clusters (86.74% taxonomic accuracy), with 116.1% agreement. The best taxonomic accuracy and agreement were found at an 8% threshold, with 258 perfect clusters (92.47%) and 278 total clusters (99.64%).

### GMYC

GMYC analysis was used to evaluate clustering outcomes using dataset-intrinsic factors rather than arbitrary preset thresholds for the delineation of molecular entities or species. GMYC analyses used the refined 270-species dataset without the resolved cryptic species. The single-threshold GMYC approach was applied to a chronogram constructed with a relaxed lognormal clock and a coalescent prior. This analysis revealed 328 GMYC entities, including 90 singletons, within a 95% confidence interval of 322–335, for an agreement of 121.48% or a 21.48% overestimation of species diversity. A total of 227 GMYC entities were perfect (taxonomic accuracy = 84.07%), and 101 entities (37.41%) belonging to 41 morphospecies were split.

Neither clustering of the refined dataset at 1–4% and final datasets at 1–5% nor GMYC analysis lumped the morphospecies. Hence, interspecific divergences were always higher than intraspecific ones, a prerequisite for molecular biodiversity assessment that is sometimes referred to as the “DNA barcoding gap” (see Fig. S2) [38], [14]. Inconsistencies between molecular entities and morphospecies were entirely due to oversplitting.

Different species hypotheses have been visualised on a subtree that also summarises the progress from morphospecies hypotheses (red bars), the analysis of nDNA (green), clustering GMYC of mtDNA (yellow / blue) and the final species hypotheses (Fig. 3). Analysis of the nDNA used a RaxML tree based on the three nuclear genes (AK, EF1α and H4). Its topology was entirely congruent to the mtDNA-based tree. The final determination was based on the relative degree of divergence and the geographical distribution pattern. The relatively shallow divergence within species 272 was interpreted as an intraspecific allopatry between two neighbouring areas. The decision to separate three cryptic species from “sp. 015 sensu lato” was based on the deep divergences between sp. 015, sp. 271, sp. 272, and sp. 273, which were furthermore negatively correlated with geographic distance.

### GMYC and objective clustering of the final dataset

The cases in which our 279 final species were not compatible with the results of the computer-based methods were examined in detail to clarify the circumstances of these “failures”. Clustering achieved its highest taxonomic accuracy at an 8% threshold, with 258 perfect clusters. At this setting, six species (spp. 041, 098, 101, 192, 229, 263) were split into two, and one species (sp. 081) was split into three; twelve species, meanwhile, were lumped. In GMYC, no lumping occurred, but 31 species (spp. 008, 011, 018, 028, 030, 041, 048, 049, 051, 081, 094, 098, 101, 114, 123, 132, 133, 150, 153, 161, 171, 172, 175, 192, 205, 225, 226, 228, 229, 240, 244, 246, 259, 261, 263, 264, 270, 272) were split into two, five species (spp. 008, 028, 051, 133, 264) into three and three species (spp. 012, 081, 205) into four. Of these split entities, 74.4% contain allopatric subclades; i.e., they represent species with geographically structured haplotype pools, where higher intraspecific divergences induce GMYC to oversplit. It is likely that denser sampling to bridge the gaps between these allopatric populations could cause the results of the final species count and GMYC to converge. Ten species (25.6%) (spp. 008, 011, 012, 030, 048, 051, 175, 225, 246, 259) contained only specimens from the same locality, and the reasons for their divergence are not always clear. There is a chance that some contain additional cryptic species, but with the available data, such a possibility is impossible to demonstrate. An example of such a doubtful species is sp. 246: a single specimen (ARC0852) diverges 7.7–9.2% from a morphologically identical cluster possessing a maximum divergence of 1.4% (average 0.6%). In other cases, however, as with sp. 259, GMYC appears to have been too sensitive and shows a tendency towards oversplitting. The species split by clustering at an 8% threshold appeared to be better justified, as they belong to allopatric populations that could be classified as “subspecies” (spp. 041, 081), are potential candidates of cryptic species (spp. 098, 263), or belong to distant allopatric populations, with no morphological differences (spp. 101, 192, 229). The species lumped by clustering at the 8% threshold usually belong to closely related sympatric species: sp. 039+sp. 109+sp. 278 from the Cyclops Mountains or sp. 115+sp. 116 from the Arfak Mountains and sp. 221+sp. 223 from Biak Island. The species in the group sp. 121+sp. 122+sp. 123 come from the Balim area but are lumped together with sp. 029, an obviously closely related species from the Cyclops Mountains.

### Success rates regional *versus* local sampling

**Figure 4:**
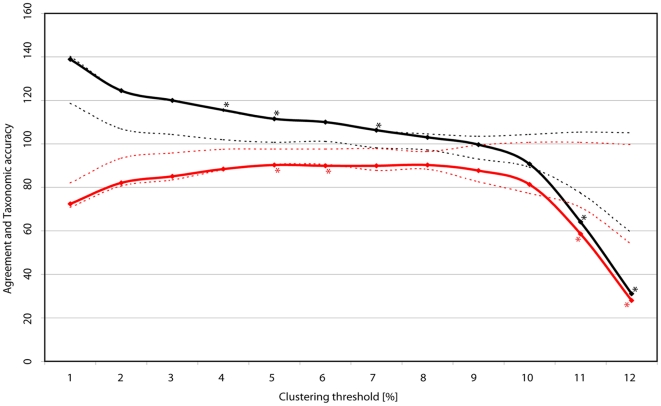
Agreement (black) and taxonomic accuracy (red) at thresholds from 1–12%. Numbers derived from the refined dataset, including 270 morphospecies. Dotted lines define 95% confidence interval of the seven areas. * = Thresholds at which clustering of the complete dataset differs statistically significant from the clustering of the areas.

To test whether there are significant differences between clustering of the regional *versus* local datasets, we calculated the 95% confidence interval of the 7 areas and checked whether the value for the complete dataset was within the 95% confidence interval; e.g., at the 1% threshold, we took the percentage value of the perfect clusters for the entire dataset and compared it with the values for each of the seven areas. We plotted the 95% confidence interval of agreement and taxonomic accuracy (Fig. 4). Values fell outside the 95% confidence interval at the 4%, 5%, 7%, 11% and 12% values for agreement and at the 5%, 6%, 11% and 12% values for taxonomic accuracy. At these thresholds, clustering of the complete dataset induced significantly different values compared with the clustering of the areas. For all other cases, this result means that there is no significant difference between the clustering of the complete dataset and the clustering of the single areas.

### ß-diversity

For the refined dataset, 259 (95.93%) species were found in just one of the seven sampling areas (Table S5). Ten species were found in two areas (spp. 018, 133, 150, 161, 192, 205, 228, 229, 244, 261), two species in three areas (spp. 001, 028) and one species in five areas (sp. 015). Out of the 279 species that were finally identified (including cryptic species), 270 (96.77%) were found in just one sampling area. Ten species were found in two areas, and one species was found in three areas. No species was found in more than three areas.

A Sørensen similarity index of 1 means that all species are shared across all areas, while an index of 0 means that no species are shared among the areas compared. The average Sørensen similarity indices for the refined and final datasets were 0.029 and 0.013, respectively, reflecting the low level of species sharing among the sampling areas. Huon, Sogeri and the Eastern Highlands had the highest values for the Sørensen similarity index, whereas Balim shared no species with any other area (Table 3). Furthermore, Sørensen similarity indices were calculated for the four localities in the Balim area (average 0.27) (Table 3) and the six localities of the Eastern Highlands (average 0.05) (Table 4).

**Table 3:** Summary of the ß-diversity between the four localities of the Balim area.

	# species	Bokondini	Habbema	Jiwika	Poga
Bokondini	11 (12)		1	3	2
Habbema	6 (6)	0.12 (0.11)		3	4
Jiwika	13 (13)	0.25 (0.24)	0.32 (0.32)		6
Poga	19 (19)	0.13 (0.13)	0.32 (0.32)	0.38 (0.38)	

Data are derived from the refined and the final (in parentheses) datasets. The upper right shows shared species, and the lower left shows the Sørensen similarity index.

**Table 4:** Summary of the ß-diversity between the six localities of the Eastern Highlands area.

	# species	Aiyura	Goroka	Haia	Mt. Michael	Okapa	Supa
Aiyura	8		2	0	0	5	0
Goroka	3	0.36		0	0	2	0
Haia	31	0.00	0.00		0	0	10
Mt. Michael	3	0.00	0.00	0.00		0	0
Okapa	13	0.24	0.25	0.00	0.00		0
Supa	17	0.00	0.00	0.21	0.00	0.00	

Data are derived from the refined dataset. The upper right shows the shared species, and the lower left shows the Sørensen similarity index.

At present, 44 species of *Trigonopterus* from New Guinea have been formally described [9], [39]. In our current dataset, three of those species (*T. nasutus*, *T. vandekampi*, *T. micros*) could be identified with confidence, while 19 others (*T. anthracinus*, *T. cribratus*, *T. curtus*, *T. densatus*, *T. dilaticollis*, *T. ephippiatus*, *T. femoralis*, *T. flavomaculatus*, *T. gibbirostris*, *T. globatus*, *T. illex*, *T. illitus*, *T. neglectus*, *T. oblongus*, *T. obnixus*, *T. pulchellus*, *T. pusillus*, *T. sejunctus*, *T. vanus*) were only tentatively named, pending a thorough taxonomic revision to include the designation of lectotypes. A final taxonomic clarification will be difficult in morphospecies that include cryptic species (e.g., *T. illex*, *T. vanus*), as the type specimens lack the necessary diagnostic characters. Most of the remaining 257 species marked by numbers here are presumably new to science.

## Discussion

*Trigonopterus* weevils are hyperdiverse across the island of New Guinea, with high α- and γ-diversity. Their ß-diversity is very high, as expected from samples that mainly originate from different mountain ranges. Despite denser sampling across the island, we still find comparably deep branching patterns between species, with an average interspecific *cox1* divergence of approximately 20% (range 12–24%). Molecular biodiversity assessment using *cox1* data is feasible, with error rates of approximately 15–20% when the number of molecular entities is compared to the number of morphospecies (refined & final dataset). Outcomes of the clustering of local *versus* regional datasets might differ with slight significance, depending on the threshold chosen. When geographically proximate localities (distance of approximately 7–52 km) were merged into one collection area, the agreement and taxonomic accuracy were lower (125%, error = 25% overestimation / 80.6%, error = 19.4% overestimation) because of the pronounced geographical genetic structure in some species. Otherwise, the performance of local and regional clustering showed mostly agreement and taxonomic accuracy using both 3% threshold clustering and GMYC (Table 2). In practice, this finding means that ß-diversity studies will probably suffer from slightly higher error rates when closer localities are studied, compared to studies of larger-scale patterns of diversity.

Distances between the localities within the Balim and Eastern Highlands areas are comparable, so the differences in their β-diversity (Tables 3 and 4) require an explanation. One possible factor might be the different geographical structure of the areas; another possibility is that differences in vegetation, or both geography and vegetation in combination, mediate the difference in ß-diversity. The mountain ranges bordering the Balim Valley (see Fig. S3) are largely covered with montane forests dominated by *Nothofagus*. The valley bottom could promote isolation but still allow colonisation of the localities that have a similar set of species. In the Eastern Highlands, the mountain structure is more complex, and most localities are located north of the watershed (except two, Haia and Supa, which are south of it). Haia and Supa support lower and mid-montane forests, while mid- and upper montane forests dominate the localities in the North. Aiyura, Goroka, and Okapa have Sørensen indices similar to the localities in the Balim area. Only two of the seven species shared between them, had intraspecific *p*-distances above 2%. However, five of the eight allopatric species in Balim had high intraspecific *p*-distances (2.05–8.79%), resulting in a lower success rate from clustering (see above). The ecology of the species collected could be an important factor as well; the mountains bordering the Balim Valley were largely untouched by human activities until quite recently, while the primary forests around Aiyura, Okapa and Goroka were reduced to patches surrounded by dominant grasslands and gardens long before the arrival of Western civilisation. Species that were somewhat adapted to these conditions could be promoted and also retain the potential for relatively recent dispersal, resulting in a lower degree of genetic structure. Additional investigations, including the study of the species' ecology and population genetics, coupled with GIS modelling, would be needed for a detailed explanation of the observed differences.

The 13 species found in more than one terrane are hardly enough to study generalities of their distribution patterns. Two of them (spp. 1, 15) had to be split into cryptic species, so their areas of distributions were markedly reduced. Of the remaining, the majority (spp. 133, 150, 161, 205, 228, 229, 244, 261) are restricted to the three areas in Papua New Guinea and include a number of montane species. Others, shared with / between the more distant areas in West New Guinea rather belong to “lowland species”, such as sp. 018, sp. 028, and sp. 192. It would be tempting to investigate the ß-diversity of localities in lowland forests, either in the Mamberamo-Sepik basin, or the Southern platform. Presumably, ß-diversity would be higher compared to the highlands.

### *Cox1* sequencing to aid taxonomy

The morphospecies count changed from the initial sorting, via a refined morphological concept, to the final species hypotheses, which combined morphological and molecular evidence. Although the absolute numbers from the initial sorting and the refined morphospecies count changed only slightly (from 274 to 270 morphospecies), the taxonomic changes were substantial. A number of species were oversplit (i.e., contained “synonyms”), but this inflation of the species count was almost exactly compensated by a failure to recognise superficially similar species. In the final step, analysis of *cox1* data stimulated the taxonomist to rethink species boundaries, accepting the existence of nine cryptic species that raised the total number to 279 species.

Because of the high number of morphologically similar species, sorting morphospecies across different localities is very difficult, even for highly trained experts. This difficulty can be clearly seen from the errors in our initial morphospecies hypotheses; the samples that were sorted at different times or came from different areas had a large number of “synonyms”, i.e., different species designation numbers were mistakenly assigned to conspecific specimens collected at different times or locations more frequently than they would be assigned to an equal number of specimens taken from the same locality and sorted at the same time. The likelihood of mistakes increased with the number of specimens as well, which was largely the result of human error and / or the occurrence of “aberrant” specimens, i.e., relatively rare individuals on the fringes of a trait range. The approach applying DNA sequence data and morphology is more reliable when large volumes of material need to be sorted, as suggested for beetles [40] and for tropical trees [41].

The current inventory of 279 species is far from complete; even for the localities sampled, not all species have been discovered. Old museum specimens representing additional species from the Cyclops Mountains and the Balim Valley are already on hand, and most of the 13 divergent clades represented by females could probably be added to the species count after males with their diagnostic characters are found (and can easily be assigned to their females using *cox1* sequences). Thus, the number of species is likely to increase with additional sampling efforts, even in the same localities. Adding more areas will further increase species discovery, and a total of more than 1000 *Trigonopterus* species is likely, as anticipated [42]. Comparing specimens side by side under the microscope or preparing provisional identification keys is not efficient when dealing with so many superficially similar species. In fact, the possibility of locating supposedly close relatives of a new specimen using DNA barcodes becomes most compelling in very large data sets. The advantages of molecular species diagnosis *versus* morphological identification also became apparent in the hyperdiverse weevil genus *Conotrachelus* [43], in a survey of ants on Madagascar [44] and in Chironomidae [45].

In addition to its use in species identification, *cox1* also contains valuable phylogenetic information. Because of its high divergence, this use is somewhat limited in *Trigonopterus*, as saturation is usually reached in groups of more than four to five closely related species. We are still confident that this effect did not affect the results of the GMYC analysis, as this program relies on the topologies and branch lengths of closely related species and their intraspecific patterns; an incorrect node at the basal position is irrelevant. At present, there are some indications for a high degree of *in situ* diversification: the clade of species 210–214 (bootstrap, BS, 86%) is endemic to the Balim area, while species 231–233 (BS 95%) are endemic to the Arfak Mountains, and the clade of species 039, 109 and 278 (BS 94%) are endemic to the Cyclops Mountains. In some cases, as with the clade of species 147–149 (BS 97%), dispersal between separate terranes seems to be involved; the species occur in the Eastern Highlands area and on the Huon peninsula. Additional, slightly more conservative markers would have to be included in a dataset to obtain a phylogeny that can resolve deeper nodes. With these data, a study investigating the extent of endemism in geological terranes could provide important insights into the evolution of a hyperdiverse group in New Guinea.

### *Cox1* sequencing for community ecology

In *Trigonopterus*, it is possible to merely rely on *cox1* sequence data to arrive at a solid starting hypothesis for comparative studies on patterns of diversity, for example ß-diversity assessment [46]. Both clustering at a 3% threshold and GMYC analyses would overestimate species diversity by 16–17%, and taxonomic error with respect to the molecular entities is approximately 14%. These data assume that the final species hypotheses are correct; however, an overestimation of species diversity, mainly involving allopatric populations, might also suggest that there are more *Trigonopterus* species in the dataset than we finally recognised. Here, more sampling and a research program focused on population genetic processes and other lines of evidence would be needed to arrive at sound taxonomic conclusions.

In any case, as soon as fifty or more *Trigonopterus* species become involved, species counts and diagnoses based on DNA barcoding become more accurate than the numbers obtained by either parataxonomists or traditional taxonomists.

### Conclusions

The present study summarises the first steps necessary to establish a new taxon for a research program on a largely unexplored fauna, building on DNA sequencing and expert taxonomic knowledge. A diverse, widespread and easy to diagnose study group prominent in all major terrestrial habitats of the Melanesian region was identified. The majority of species possess clear morphological characters and only a minor proportion (3.3%) had to be classified as cryptic species at a later stage. Both objective clustering at variable thresholds (3% to 8%) and a GMYC analysis outperform the parataxonomist. DNA barcoding proves to be an excellent tool for surveying both locally and supraregionally but may exhibit a slight drop in performance when localities 7–52 km away are included.

The tools available to identify *Trigonopterus* weevils thus allow researchers to overcome the taxonomic impediment. The advantages of weevils over butterflies or vertebrates commonly used in rapid biodiversity assessment (RAP) surveys should be apparent; collecting is relatively easy, and weevils are present in most primary forests of Melanesia from sea level to subalpine grasslands. Even forest remnants of limited size likely harbour a large number of species. As shown above, endemism is high, and the chance that an isolated forested hill has its own set of endemic species is good. If it is our purpose to protect the biodiversity of Melanesia, *Trigonopterus* weevils are surely a valuable part of that biodiversity, both in the numbers of species and in terms of quality as an indicator. The tools are ready; it is now up to conservationists to use them.

## Supporting Information

Figure S1 **Maximum likelihood tree based on** ***cox1*** **sequences of 1,002 specimens of** ***Trigonopterus*** **.** Seven outgroup representatives are included. Specimens come from seven areas of New Guinea. Clusters correlate with the assignment of 270 morphospecies given to the right and nine additional cryptic species marked in red. The dataset in nexus format is available from the authors.
(TIF)

Figure S2 **Distribution of all average intra- and interspecific distances based on the refined dataset.**
(TIF)

Figure S3 **Balim area showing all sampling points at the four localities.**
(TIF)

Table S1 **Overview of field work in each collecting area.** ^1^ = sampling points with a minimum distance of 7 km are counted as separate localities.
(DOC)

Table S2 **Primers used in this study.**
(DOC)

Table S3 **Average intraspecific p-distances of** ***Trigonopterus*** **that are higher than 3%.** Based on the entire refined dataset. EHL = Eastern Highlands. The term “subspecies” describes allopatric populations with distinct but minor morphological differences.
(DOC)

Table S4 **Clustering of four localities in the Balim area.**
(DOC)

Table S5 **Summary of the ß-diversity between the seven sampled areas in New Guinea.** Data are derived from the refined dataset (final dataset in parentheses). The upper right shows species shared between areas; the lower left shows the Sørensen similarity index. Numbers following area names indicate the number of species encountered.
(DOC)
