# Supplementary figures and images for: DNA Barcoding for Community Ecology - How to Tackle a Hyperdiverse, Mostly Undescribed Melanesian Fauna
Source: PLoS One. 2012 Jan 13;7(1):e28832. doi: 10.1371/journal.pone.0028832 (PMC3258243; doi:10.1371/journal.pone.0028832)

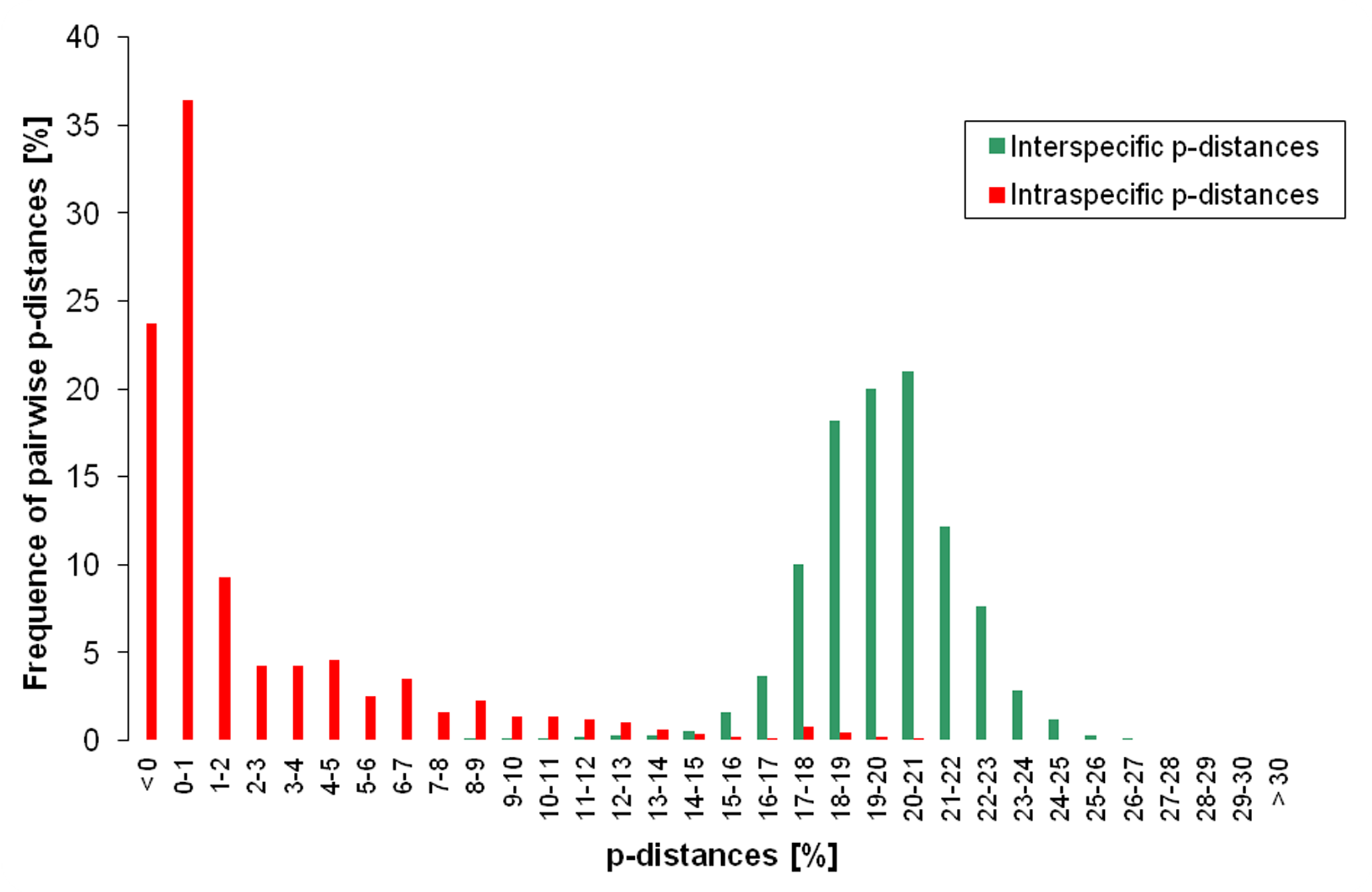

Supplement: Figure S2 — Distribution of all average intra- and interspecific distances based on the refined dataset. (TIF) [file pone.0028832.s002.tif]

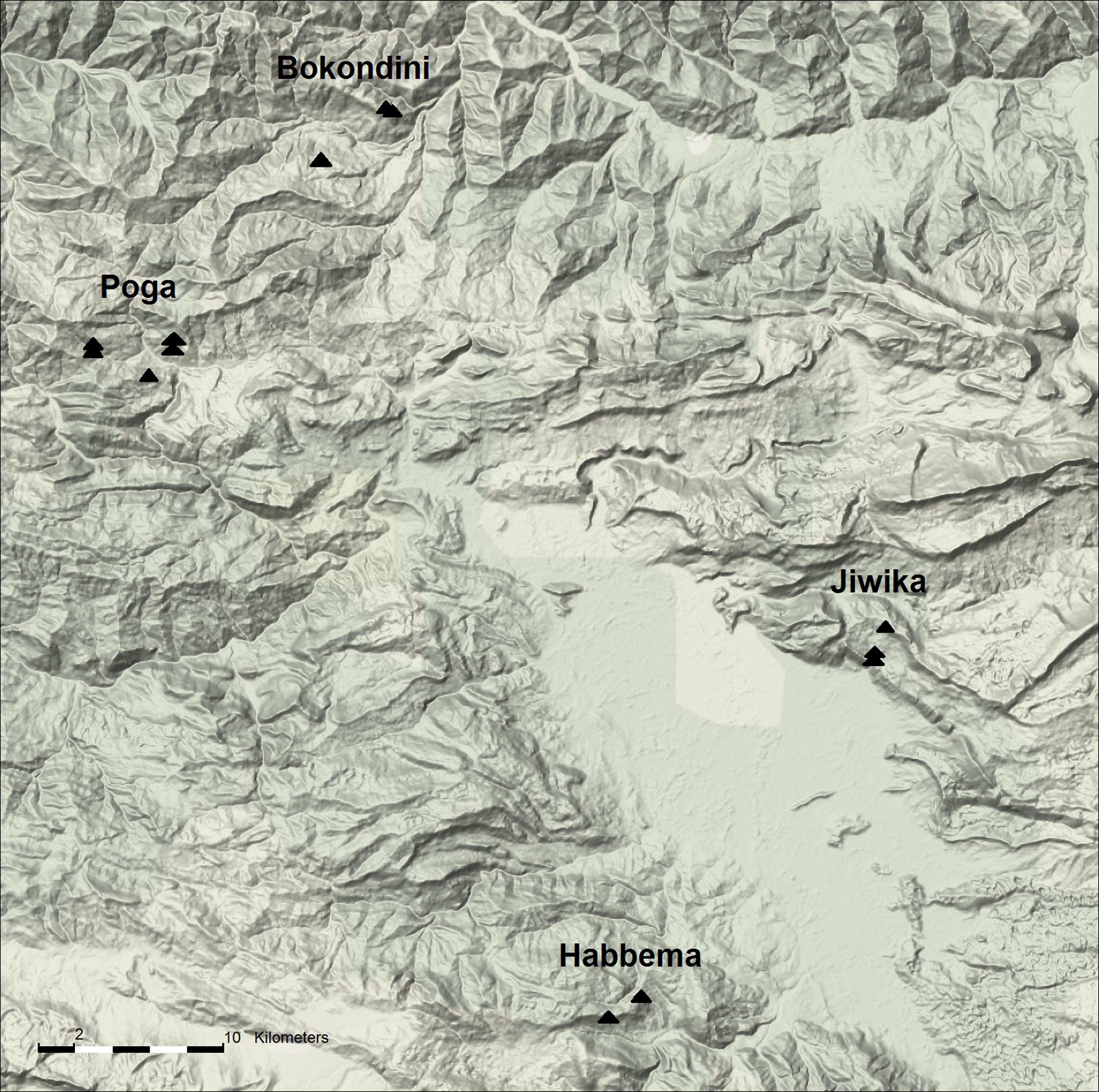

Supplement: Figure S3 — Balim area showing all sampling points at the four localities. (TIF) [file pone.0028832.s003.tif]
