# Supplementary material for: DNA Barcoding for Community Ecology - How to Tackle a Hyperdiverse, Mostly Undescribed Melanesian Fauna
Source: PLoS One. 2012 Jan 13;7(1):e28832. doi: 10.1371/journal.pone.0028832 (PMC3258243; doi:10.1371/journal.pone.0028832)
Supplement: Table S1 — Overview of field work in each collecting area. 1 = sampling points with a minimum distance of 7 km are counted as separate localities. (DOC) [file pone.0028832.s004.doc]

| Area | Arfak | Balim | Biak | Cyclops | EHL | Huon | Sogeri |
| --- | --- | --- | --- | --- | --- | --- | --- |
| Number of localities¹ | 2 | 4 | 2 | 2 | 5 | 3 | 2 |
| Days of field work | 6 | 10 | 3 | 7 | 12 | 8 | 5 |
| Number of litter samples | 9 | 6 | 2 | 16 | 7 | 7 | 0 |
| Elevational range | 220-1870m | 1655-2770m | 50-200m | 300-1520m | 750-2800m | 480-1710m | 500-920m |
