# Supplementary material for: DNA Barcoding for Community Ecology - How to Tackle a Hyperdiverse, Mostly Undescribed Melanesian Fauna
Source: PLoS One. 2012 Jan 13;7(1):e28832. doi: 10.1371/journal.pone.0028832 (PMC3258243; doi:10.1371/journal.pone.0028832)
Supplement: Table S2 — Primers used in this study. (DOC) [file pone.0028832.s005.doc]

| Gene | Location | Primer | Direction | Sequence (5' -> 3') | PCR protocol | References |
| --- | --- | --- | --- | --- | --- | --- |
| Cox1 (Cytochrome c oxidase 1) | mitochondrial | LCO1490-JJ | FOR | CHACWAAYCATAAAGATATYGG | D:94°C for 30 sec; A:47(52)°C for 40 sec; E:72°C for 60 sec | *Astrin & Stüben, 2008* |
|  |  | HCO2198-JJ | REV | AWACTTCVGGRTGVCCAAARAATCA | 5 cycles 47°C, 30 cycles 52°C | *Astrin & Stüben, 2008* |
| AK (Arginine kinase) | nuclear | AK183F | FOR | GATTCTGGAGTCGGNATYTAYGCNCCYGAYGC | D:94°C for 30 sec; A:53°C for 30 sec; E:72°C for 60 sec | *Wild & Maddison, 2008* |
|  |  | AK939R | REV | GCCNCCYTCRGCYTCRGTGTGYTC | 35 cycles | *Wild & Maddison, 2008* |
| EF1α (Elongation factor 1α) | nuclear | efs372 | FOR | CTGGTGAATTTGAAGCYGGTA | D:94°C for 30 sec; A:58-42°C for 30 sec; E:72°C for 60 sec | *McKenna et al, 2005* |
|  |  | efa754 | REV | CCACCAATTTTGTAGACATC | ΔT -2°C every 3 cycles (58-44°C); 18 cycles 42°C | *Normark et al, 1999* |
| H4 (Histone 4) | nuclear | H4F2s | FOR | TSCGIGAYAACATYCAGGGIATCAC | D:94°C for 30 sec; A:49°C for 30 sec; E:72°C for 60 sec | *Pineau et al, 2004* |
|  |  | H4F2er | REV | CKYTTIAGIGCRTAIACCACRTCCAT | 35 cycles | *Pineau et al, 2004* |
