# Supplementary material for: DNA Barcoding for Community Ecology - How to Tackle a Hyperdiverse, Mostly Undescribed Melanesian Fauna
Source: PLoS One. 2012 Jan 13;7(1):e28832. doi: 10.1371/journal.pone.0028832 (PMC3258243; doi:10.1371/journal.pone.0028832)
Supplement: Table S3 — Average intraspecific p-distances of Trigonopterus that are higher than 3%. Based on the entire refined dataset. EHL = Eastern Highlands. The term “subspecies” describes allopatric populations with distinct but minor morphological differences. (DOC) [file pone.0028832.s006.doc]

| **species** | **intraspecific p-distance** | **remarks** |
| --- | --- | --- |
| *Trigonopterus* sp. 001 | 19.49% | sp.1 + 3 cryptic species (sp. 270, sp. 275, sp. 276). |
| *Trigonopterus* sp. 086 | 14.21% | sp.86 + 1 cryptic species (sp. 274). |
| *Trigonopterus* sp. 098 | 11.40% | Probably sympatric, although from different hills. Morphology identical. Possibly cryptic species. |
| *Trigonopterus* sp. 015 | 9.46% | sp.15 + 3 cryptic species (sp. 271-273). Minor differences in aedeagi and external morphology. |
| *Trigonopterus* sp. 229 | 8.79% | Allopatry Huon / Sogeri. Morphology identical. |
| *Trigonopterus* sp. 081 | 8.79% | Allopatry Bokondini / Poga / Jiwika-Habbema. Minor morphological differences (e.g. punctation of pronotum more or less dense). “Subspecies”. |
| *Trigonopterus* sp. 041 | 7.79% | Allopatry Sentani / Angkasa indah. Minor morphological differences (e.g. genitalia; surface dull or rather shining). “Subspecies”. |
| *Trigonopterus* sp. 101 | 7.57% | Allopatry Ransiki / Mokwam. No morphological differences, but ARC0797 / ARC0798 are females. |
| *Trigonopterus* sp. 263 | 7.50% | Allopatry Varirata / Moroka. Aedeagus of Varirata-male with longer apodemes and longer flagellum. Either cryptic species or "subspecies". |
| *Trigonopterus* sp. 132 | 7.29% | Allopatry Aiyura / Okapa. No morphological differences, but Okapa-specimen is a female. |
| *Trigonopterus* sp. 247 | 7.09% | sp.247 + 1 cryptic species (sp. 279). Sympatry. |
| *Trigonopterus* sp. 226 | 6.00% | Allopatry Jiwika / Poga. Minor morphological differences present. “Subspecies”. |
| *Trigonopterus* sp. 012 | 5.60% | Partly sympatry (Sentani), partly allopatry (Sentani / Angkasa indah). No morphological differences, but genitalia of this group poor in characters. |
| *Trigonopterus* sp. 205 | 5.43% | Allopatry Aiyura / Goroka/ Pindiu-Mindik. No morphological differences except Minik-specimens with deeply impressed striae. Recent dispersal into Huon-area? |
| *Trigonopterus* sp. 150 | 5.41% | sp.150 + 1 probably sympatric, cryptic species (sp. 269). |
| *Trigonopterus* sp. 192 | 5.24% | Allopatry Biak / Huon. No morphological differences. |
| *Trigonopterus* sp. 264 | 5.17% | Allopatry Varirata / Moroka. Specimen from Moroka is a female. |
| *Trigonopterus* sp. 228 | 4.90% | Allopatry EHL / Huon. No morphological differences. |
| *Trigonopterus* sp. 133 | 4.51% | Allopatry Sogeri / Huon. No morphological differences. |
| *Trigonopterus* sp. 171 | 4.46% | Allopatry Jiwika / Bokondini. Minor morphological differences present in mesofemur. “Subspecies”. |
| *Trigonopterus* sp. 261 | 4.26% | Allopatry EHL / Sogeri. No morphological differences. |
| *Trigonopterus* sp. 244 | 3.58% | Allopatry EHL / Sogeri. Elytra of EHL with denser punctuation. |
| *Trigonopterus* sp. 049 | 3.53% | Allopatry Sentani / Angkasa indah. Minor morphological differences (e.g. genitalia; denticle on rostrum). “Subspecies”. |
| *Trigonopterus* sp. 259 | 3.34% | Sympatry. Unique female divergent. No apparent morphological difference. |
| *Trigonopterus* sp. 175 | 3.23% | Sympatry. Doubtful: One cluster consists of males, the other (ARC0351 etc) of females. No apparent morphological difference. |
