# Supplementary material for: DNA Barcoding for Community Ecology - How to Tackle a Hyperdiverse, Mostly Undescribed Melanesian Fauna
Source: PLoS One. 2012 Jan 13;7(1):e28832. doi: 10.1371/journal.pone.0028832 (PMC3258243; doi:10.1371/journal.pone.0028832)
Supplement: Table S4 — Clustering of four localities in the Balim area. (DOC) [file pone.0028832.s007.doc]

| analyzed dataset | number morpho- species | number cluster /  entities | agreement  [%] | number perfect fit | taxonomic  accuracy [%] | lumped  cluster | split  cluster |
| --- | --- | --- | --- | --- | --- | --- | --- |
| Jiwika | 13 | 14 | 107.7 | 12 | 92.3 | 0 | 2 |
| Poga | 19 | 19 | 100.0 | 19 | 100.0 | 0 | 0 |
| Bokondini | 11 | 12 | 109.1 | 10 | 90.9 | 0 | 2 |
| Habbema | 6 | 6 | 100.0 | 6 | 100.0 | 0 | 0 |
