# Supplementary material for: DNA Barcoding for Community Ecology - How to Tackle a Hyperdiverse, Mostly Undescribed Melanesian Fauna
Source: PLoS One. 2012 Jan 13;7(1):e28832. doi: 10.1371/journal.pone.0028832 (PMC3258243; doi:10.1371/journal.pone.0028832)
Supplement: Table S5 — Summary of the ß-diversity between the seven sampled areas in New Guinea. Data are derived from the refined dataset (final dataset in parentheses). The upper right shows species shared between areas; the lower left shows the Sørensen similarity index. Numbers following area names indicate the number of species encountered. (DOC) [file pone.0028832.s008.doc]

|  | # species | Arfak | Balim | Biak | Cyclops | EHL | Huon | Sogeri |
| --- | --- | --- | --- | --- | --- | --- | --- | --- |
| Arfak | 37 (38) |  | 0 (0) | 1 (0) | 2 (1) | 1 (0) | 0 (0) | 1 (0) |
| Balim | 36 (37) | 0.00 (0.00) |  | 0 (0) | 0 (0) | 0 (0) | 0 (0) | 0 (0) |
| Biak | 16 (16) | 0.04 (0.00) | 0.00 (0.00) |  | 1 (0) | 1 (0) | 1 (1) | 1 (0) |
| Cyclops | 54 (54) | 0.04 (0.02) | 0.00 (0.00) | 0.03 (0.00) |  | 1 (0) | 2 (1) | 2 (1) |
| EHL | 57 (58) | 0.02 (0.00) | 0.00 (0.00) | 0.03 (0.00) | 0.02 (0.00) |  | 4 (3) | 3 (2) |
| Huon | 55 (55) | 0.00 (0.00) | 0.00 (0.00) | 0.03 (0.03) | 0.04 (0.02) | 0.07 (0.05) |  | 4 (4) |
| Sogeri | 33 (33) | 0.03 (0.00) | 0.00 (0.00) | 0.04 (0.00) | 0.05 (0.02) | 0.07 (0.04) | 0.09 (0.09) |  |
